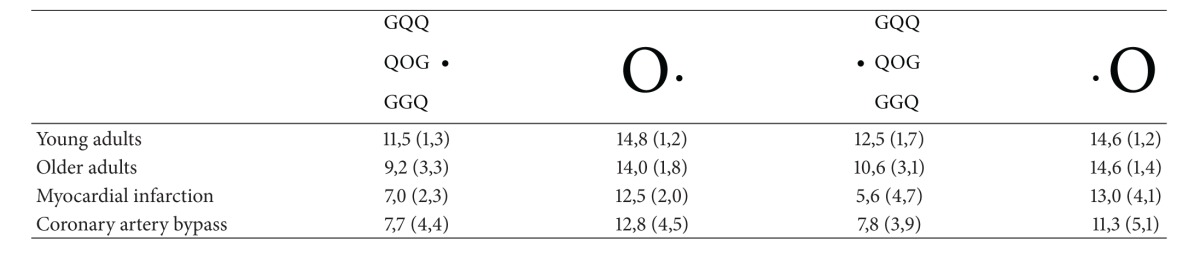# Effects of Age and Cardiovascular Disease on Selective Attention

**DOI:** 10.1155/2013/185385

**Published:** 2013-12-25

**Authors:** Sylvie Chokron, Gérard Helft, Céline Perez

**Affiliations:** ^1^Laboratoire de Psychologie de la Perception, CNRS, UMR 8158 & Université Paris-Descartes, 75006 Paris, France; ^2^Unité Fonctionnelle Vision et Cognition & Service de Neurologie, Fondation Ophtalmologique Adolphe de Rothschild, 25 Rue Manin, 75019 Paris, France; ^3^Institut de Cardiologie, Hôpital de la Salpêtrière, 75013 Paris, France

## Abstract

In order to study the effect of normal aging and cardiovascular disease on selective attention, a letter-identification task was proposed to younger and older healthy adults as well as patients with a recent myocardial infarction or a recent coronary artery bypass grafting. Participants had to detect either a big stimulus or a small one surrounded by flanking letters. The stimuli were displayed horizontally, either in the left (LVF) or in the right visual field (RVF). The interaction between the type of stimulus and the hemifield of presentation reached significance in all groups except in patients who underwent a coronary artery bypass. Only young normal adults showed the expected significant RVF advantage when detecting big stimuli and an LVF advantage when detecting small stimuli surrounded by flankers. In older control adults and in patients with myocardial infarction, the RVF advantage for the condition with selective attention vanished. In patients who underwent a coronary artery bypass, reaction times were increased and no hemispheric specialization for selective attention emerged. The results are discussed with regard to the hypothesis of a Hemispheric Asymmetry Reduction in Older Adults (HAROLD model) and to the presence of cognitive dysfunction consecutive to cardiovascular disease.

## 1. Introduction

The concept of selective attention usually refers to the ability to focus on areas of visual space to facilitate target detection [1]. Using a visual detection paradigm adapted from LaBerge and Buchsbaum [2], and previously shown to activate the pulvinar [3] we demonstrated that when selective attention is required to identify a visual target surrounded by flankers, reaction times (RTs) are shorter in the right than in the left visual field [4, 5], thus confirming a left hemisphere (LH) advantage for filtering irrelevant information and analysing the local features of a visual scene [6, 7]. Conversely, RTs are found to be shorter in the left visual field (LVF) than in the right visual field (RVF) when the to-be-identified target is presented alone and required less filtering activity, that is, less selective attention. These data were obtained in young healthy right-handed adults (average age, 28.4 years in Chokron et al. [4]), but as several studies have hypothesized, aging may modify both selective attention processes and the pattern of cerebral lateralization [8].

Cabeza et al. [9] measured prefrontal activation in younger and older adults performing memory tasks. They found that high-functioning older adults showed strong bilateral prefrontal activations whereas young subjects involved only a smaller prefrontal circuit in the right hemisphere and proposed that in aging subjects, there could be a Hemispheric Asymmetry Reduction in Older Adults (HAROLD model) for some cognitive functions. They thus contended that high-functioning older adults compensate for age-related neural decline through a compensatory reorganization of their neurocognitive networks.

In addition, an impairment of central nervous system function is thought to underlie much of the cognitive decline that often accompanies advancing age. Histological changes, though not uniform, are widespread in the aged brain [10] and it has been commonly held that the psychological effects of age are due to a progressive diffuse loss of cerebral tissue [11]. However, when normal elderly individuals are actually compared to patients with documented diffuse brain disease, their psychological test profiles are actually very different [12, 13]. Some researchers have suggested that whatever the anatomical distribution of the underlying structural and physiological changes that occur in old age, certain major regions of the brain may be more affected by aging than are others. In particular, the right hemisphere has been singled out as being particularly sensitive to the deleterious effects of aging [8, 14, 15]. With regard to this work, the apparently greater decline in spatial abilities in the elderly appears to be the consequence of age having a disproportionately greater effect in right-hemispheric function than it does on left-hemispheric function. If there is a modification of the pattern of hemispheric specialization and/or if there is a specific decline of the right hemisphere in the elderly, we should observe an effect of aging on the abovementioned pattern of hemispheric specialization for selective attention previously found in young adults.

On the other hand, normal aging is often associated with either hypertension and/or cardiovascular disease. The presence of a cardiovascular disease and hypertension is usually not controlled when studying the effect of aging on cognitive function. However, several recent data support the hypothesis that vascular disease including hypertension and myocardial infarction is predictive of poor cognitive function (see Prince [16] and de la Torre [17] for review) but the nature and extent of these deficits remain unclear. As a matter of fact, most of the studies including the Framingham Heart Study [18, 19] have investigated the role of cardiovascular risk on memory tasks but attentional processes, which might decline before memory and verbal functions [20], had not been evaluated in these patients.

The present study was thus designed to study the effect of both normal aging and vascular disease on selective attention as well as on the hemispheric pattern of specialization for these processes. For this purpose, we compared the performance of younger and older adults free from any cardiovascular disease to age-matched patients who had underwent a myocardial infarction and suffered from a cardiovascular disease (hypertension, pectoris angina) or a coronary bypass.

## 2. Methods

### 2.1. Subjects

Thirteen young adults (6 men, 7 women, average age: 28,8 years; SD = 1.92), twenty-three older adults (13 men, 10 women, average age: 56,5 years; SD = 3.79), eight patients who had suffered from a myocardial infarction in the last 2 months (7 men, 1 woman, average age: 59,3 years; SD = 2.09), and nine patients who underwent a coronary bypass in the last 2 months (3 men, 6 women, average age: 65,3 years; SD = 2.46) volunteered to participate in the study. They all had normal-to-corrected vision and left-to-right reading habits and used the Roman alphabet. All participants were right-handed, right-footed, and right-eyed as measured by a laterality questionnaire [21]. Although the coronary bypass group was older on average than the two other older adults groups (healthy and myocardial groups), the age averages did not significantly differ from each other.

### 2.2. Procedure

Subjects sat in a comfortable chair, directly in front of the middle of the computer screen and, at a distance of 57 cm and looked at stimuli positioned horizontally at 2° to the right or to the left of the central fixation point. During the whole experiment, subjects had to visually fixate a dot corresponding to the centre of the screen and were presented with 16 blocks, each of eight trials. Between each block, there was a 20-second period of rest while the screen was gray and subjects could close their eyes if they wished to.

**Figure 1:**
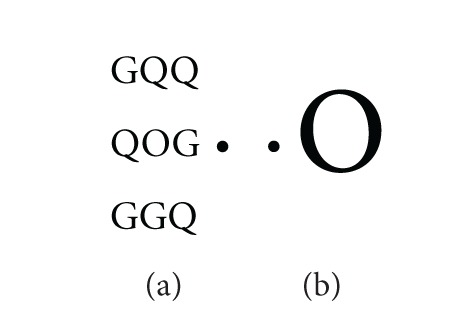
Examples of experimental conditions: (a) the task is to detect the small O surrounded by flankers (condition with selective attention); (b) the task is to detect the big O presented alone (condition without selective attention).

Subjects were presented with 128 stimuli, 64 in the left visual field and 64 in the right visual field. The stimuli were the letter O, the letter C, or the digit zero (0). The stimulus appeared either alone as a big character or as a small character surrounded by eight other similar letters (G and Q) (see Figure 1). The overall size of the stimuli was controlled so that the big letters were of the same dimensions as the patterns of small letters surrounded by the flankers, that is, 19 mm wide × 22 mm high.

The participants had to detect the target (the letter O) that could be presented as a big letter, alone or as a small letter surrounded by flankers (the letters G and Q as in Figure 1). On some trials, the participants were presented with a C or a 0 (alone or surrounded by flankers) and have to ignore these stimuli.

In case of the target appearing in the left visual field, subjects had to click on the left button of the mouse, whereas they had to press on the right for a right-sided target. Each display was flashed for 150 ms. When the subject responded, a 2000 ms intertrial interval began. If the subject did not respond, a 1000 ms delay ensued. For detailed description of the protocol, see Tabert et al. [22].

### 2.3. Data Analysis

For each subject, each type of stimulus (big presented alone or small surrounded by flankers), and each side of target location (left or right), we recorded the reaction times in milliseconds, as well as the number of correct detections (maximum 16 per stimulus type) that were evaluated by a three-way analysis of variance with group (young adults, older adults, patients with myocardial infarction, and patients who underwent a coronary bypass) as a between factor and type of stimulus (alone or surrounded by flankers) and visual field (left or right) as within factors.

## 3. Results

### 3.1. Young Adults

**Figure 2:**
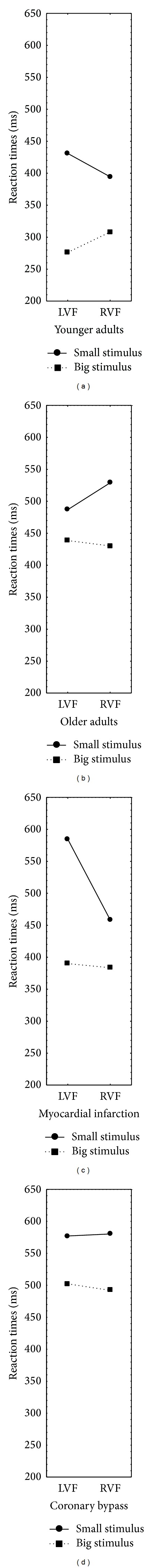
Reaction times (ms) for each group (young adults, older adults, patients with myocardial infarction, and patients with coronary artery bypass), for each stimulus (small surrounded by flankers, small presented alone), and each visual field of presentation (left or right).

As previously demonstrated [4, 5], single targets led to shorter RTs (*m* = 292 ms, SD = 60) than small targets surrounded by flankers (*m* = 412.5 ms, SD = 66) (*F*1–11 = 123.16; *P* < .00001) (Table 1). We also confirmed the presence of a significant interaction between the stimulus type and the visual field of presentation (*F*1–11 = 18.55; *P* < .0012). As presented in Table 1 and Figure 2, for big targets presented alone, RTs were shorter when the stimulus was presented in the left than in the right visual field. On the contrary, for small targets surrounded by flankers, RTs were shorter when the stimulus was presented in the right than in the left visual field. We thus confirmed the hemispheric specialization for selective attention with a right hemisphere (RH) superiority for visual detection and a left hemisphere (LH) superiority for selective attention.

In terms of accuracy, we found as expected a significant effect of the stimulus type (*F*1–12 = 54.85, *P* < .0001) with a higher accuracy rate for big stimuli presented alone than for small stimuli presented surrounded by flankers (see Table 2).

### 3.2. Older Adults

As expected, single targets led to shorter RTs (*m* = 486 ms, SD = 112) than small targets surrounded by flankers (*m* = 528 ms, SD = 116) (*F*1–11 = 23.38; *P* < .001). As previously shown, a significant interaction between the stimulus type and the visual field of presentation emerged (*F*1–23 = 6.59; *P* < .05). However, conversely to what was found for younger subjects, the visual field of presentation had a significant effect only for small stimuli surrounded with flankers (see Table 1 and Figure 2). In this case and conversely to what was found in younger subjects, RTs were shorter when the stimulus was presented in the left than in the right visual field. On the contrary, for big stimuli presented alone, RTs tended to be shorter when the stimulus was presented in the right than in the left visual field, but conversely to what was observed in younger adults, this difference was not statistically significant (see Table 1 and Figure 2). We thus surprisingly found in older adults a right (instead of the expected left) hemisphere superiority for selective attention and no hemispheric superiority for global visual detection.

In terms of accuracy, we found as in younger adults a significant effect of the stimulus type (*F*1–22 = 6.61, *P* < .02) with a higher accuracy rate for big stimuli presented alone than for small stimuli surrounded by flankers (see Table 2). In addition, the visual field of presentation had a significant effect on performance (*F*1–22 = 8.38, *P* < .00001) with better accuracy in the right than in the left visual field (see Table 2).

### 3.3. Patients Who Suffered from a Myocardial Infarction

As shown in the previous groups, there was a significant effect of the stimulus type (*F*1–7 = 68.43, *P* < .0001) with shorter RTs for big targets presented alone than for small targets surrounded by flankers (see Table 1). In this group, a statistically significant effect of the visual field emerged (*F*1–7 = 11.70, *P* < .01) with shorter RTs in the right than in the left visual field (see Table 1). Again, the interaction between the stimulus type and the visual field of presentation proved to be significant (*F*1–7 = 6.012, *P* < .05), but conversely to what was found in the other groups (younger and older control adults) the visual field of presentation had a significant effect only for small targets surrounded by flankers and not for big targets presented alone (see Table 1 and Figure 2). As in the young adults group, in terms of RTs we found here an LH superiority for selective attention, but not the expected RH specialization for global visual detection.

In terms of accuracy, we found as for the other groups an effect of the stimulus type (*F*1–7 = 11.88, *P* < .002) with better performance for big rather than for low stimuli (see Table 2).

### 3.4. Patients Who Underwent a Coronary Bypass

We confirmed in this group the significant effect of the stimulus type (*F*1–7 = 68.43, *P* < .0001) with shorter RTs for big targets presented alone than for small targets surrounded by flankers (see Table 1). However, in this group, the interaction between the stimulus type and the visual field of presentation did not reach significance (*F*1–8 = .25, ns). Conversely to what was found in the other groups in terms of RTs, no visual field advantage, that is, no hemispheric, specialization emerged whatever the stimulus type presented (big stimulus presented alone or small stimulus surrounded by flankers) (see Table 1 and Figure 2).

In terms of accuracy, we found as expected an effect of the stimulus type (*F*1–8 = 18.31, *P* < .02) with better performance for big rather than for low stimuli (see Table 2).

In summary, younger adults presented the expected hemispheric specialization for selective attention (LH superiority for small stimuli and RH superiority for big stimuli). In older subjects, with associated heart disease or not, no hemispheric specialization was observed for the global visual detection condition, whereas in the condition, where selective attention was required, subjects with myocardial infarction exhibited a left hemisphere superiority as younger controls, whereas older healthy subjects exhibited the reversed specialization (RH specialization for selective attention).

### 3.5. Between-Group Comparison

The ANOVA performed on group (younger adults, older adults, patients with myocardial infarction, and patients who underwent a coronary bypass) as a between factor and type of stimulus (alone or surrounded by flankers) and visual field (left or right) as within factors revealed a significant effect of the group (*F*3–49 = 9.53; *P* < .00005) as well as a significant interaction between the group and the visual field of presentation (*F*3–49 = 3.95; *P* < .02) and a significant interaction between the group, the visual field, and the type of stimulus (see Table 1 and Figure 2). This complex interaction stemmed from the fact that the stimulus type and the visual field of presentation had a specific effect in each group as illustrated in Figure 2.

We present below the comparison between the different groups.

#### 3.5.1. Comparison between Younger and Older Control Adults

Whatever the stimulus type and the visual field of presentation, younger normal adults RTs were significantly faster than older normal adults RTs (see Table 1) (*F*1–34 = 16.60, *P* < .0005). In addition, when younger and older adults were compared, we found a significant interaction between the groups and the stimulus type (*F*1–34 = 4.21; *P* < .05). This interaction stemmed from the fact that older adults' RTs for big targets presented alone were significantly slower than younger adults' in the same condition (see Figure 2). Finally, a significant interaction emerged also between the group, the visual field of presentation, and the stimulus type, (*F*1–34 = 15,60; *P* < .0005) due to the fact that, as mentioned above, the stimulus type and the visual field of presentation interacted in a different way for each group (see Figure 2).

#### 3.5.2. Comparison between Older Control Adults and Patients with Myocardial Infarction

When comparing these two groups, a significant interaction between the group and the visual field of presentation emerged (*F*1–30 = 9.21; *P* < .005) as well as between the group and the stimulus type (*F*1–30 = 4.7; *P* < .05). As shown in Table 1 and Figure 2, patients with myocardial infarction had much shorter RTs in the right than in the left visual field for small stimuli surrounded by flankers, whereas in older controls the reverse was observed for small targets. For big targets, results of the two groups were comparable (see Figure 2).

In addition, as presented in Figure 2, whereas controls' RTs (*m* = 507 ms) were shorter than patients (*m* = 522 ms) in the condition with selective attention (small target surrounded by flankers), the reverse was observed in the condition without selective attention (big target presented alone) where patients with myocardial infarction (*m* = 387 ms) were faster than controls of the same age (*m* = 434 ms) (see Table 1 and Figure 2).

#### 3.5.3. Comparison between Older Control Adults and Patients with Coronary Artery Bypass

Patients with coronary artery bypass grafting did not significantly differ from controls of the same age (*F*1–31 = 3.64; *P* = .065) (see Figure 2).

#### 3.5.4. Comparison between Patients with Myocardial Infarction and Patients with Coronary Artery Bypass

Patients with coronary artery bypass were found to significantly differ from patients with myocardial infarction (*F*1–15 = 4.37; *P* = .05). This effect stemmed from the fact that patients with coronary artery bypass were significantly slower than patients with myocardial infarction (see Figure 2). In addition, we found a significant interaction between the group and the visual field of presentation (*F*1–15 = 5.16; *P* < .05) due to the fact that only in patients with myocardial infarction the visual field of presentation significantly affects the RTs for small stimuli surrounded by flankers (see Table 1 and Figure 2).

## 4. Discussion

The present study was designed to investigate the effect of age and of vascular disease on both selective attention performance and hemispheric specialization for such processes. For this purpose, we submitted two groups of healthy adults participants (aged 28.8 and 56.5 years on average) as well as cardiovascular patients to a lateralized visual detection task which implies or not selective attention. First of all, we confirmed in all groups shorter reaction times when detecting big targets presented alone rather than small targets surrounded by flankers. Hemispheric specialization for selective attention was confirmed, but only in younger adults as initially shown [4]. In this group we found, as previously demonstrated, a left visual field advantage, that is, a right hemisphere (RH) specialization for visual detection, which does not imply any filtering process, and a right visual field advantage, that is, a left hemisphere (LH) specialization for visual selective attention. However, in older control participants instead of the expected left hemisphere superiority for selective attention we found a right hemisphere advantage. Moreover, in patients who recently underwent coronary artery bypass grafting, no significant interaction emerged between the visual field of presentation and the experimental condition (with or without selective attention).

Below, we discuss the effect of aging and of cardiovascular disease on selective attention processing.

### 4.1. Effect of Aging on Selective Attention and Hemispheric Specialization

As mentioned in the introduction, the present finding of a hemispheric specialization for global and local visual processing only in younger adults confirms the hypothesis of a change in hemispheric specialization with aging. As cited in the introduction, some authors have proposed that the decline found in selective attention processes with normal aging [23, 24] may be induced by a loss of hemispheric specialization for such processes. In addition, conversely to younger adults who showed the expected LH (RVF) advantage for selective attention both in terms of RT and accuracy, older adults exhibited this pattern but only in terms of accuracy. Indeed in terms of RT, they showed an RH (LVF) advantage for this condition. This opposite pattern of results could explain the contradictory results found in several recent studies investigating the effect of aging on selective attention. For example, using a spatial orienting task, in which participants responded to attended and unattended peripheral targets while recording event-related potentials (ERPs) to both targets and attention-directing spatial cues, Nagamatsu et al. [25] found that, coherent with the present results, seniors also had significantly higher error rates for targets presented in the left versus right visual field during a visual detection task of attended versus unattended stimuli.

However, other authors did not find any difference in adult performance during visual detection tasks regarding the participant's age. But, as suggested by the present study as well as by other authors [26–28] apparently contradictory findings may stem from the characteristics of the task, its attentional load, and the measure of the performance (RT or accuracy).

In addition, to the effect of age on hemispheric specialization for selective attention, the present findings also suggest an effect of the presence and type of associated diseases such as cardiac pathology on attentional performance in senior participants.

### 4.2. Effect of Cardiovascular Disease on Selective Attention and Hemispheric Specialization

First of all, the type of cardiac disease (myocardial infarction versus coronary bypass) had a significant effect on the reaction times in a letter-detection task. As a matter of fact, patients who had a myocardial infarction exhibited shorter reaction times than normal controls of the same age when detecting visual target in the condition without selective attention. On the contrary, in the condition with selective attention, these patients were found to be slower than their normal peers. These shorter reaction times in patients with myocardial infarction compared to controls could be the consequence of either the postinfarction stress that could enhance their responsiveness in a simple detection task or to the intrinsic characteristics of the personality of such patients often described as type-A personality [29].

As recently reviewed by Cohen and Mather [30], heart failure is a growing epidemic with an estimated 5 million Americans suffering from this condition. Several clinical trials have demonstrated a high correlation between congestive heart failure (CHF) and cognitive impairment. The severity of cognitive impairment correlates positively with the degree of CHF. According to Polidori et al. [31], the underlying mechanism for cognitive impairment remains unclear but appears to be related to cerebral hypoperfusion and impaired cerebral reactivity with selective impairment of verbal memory and attention domains. This explains why the present task which involves both letters and selective attention may be particularly sensible to cardiac disease in older subjects. Furthermore, according to several authors (see for review [30, 31]), cognitive dysfunction represents one aspect of frailty, a novel concept that encompasses a range of clinical conditions that results in functional impairment in patients with heart failure. According to recent reviews in this field, cognitive impairment seems to be a common and predictable effect of cardiovascular disease in the elderly [18, 19] and this could contribute with social and behavioral problems to decreased compliance to prescribed therapy and increased hospital readmissions. In addition, according to Polidori et al. [31], the pathophysiology of cardiac failure in cognitive impairment should be addressed in light of possible preventive strategies against the onset of AD. Regarding the lack of studies investigating the link between cardiovascular disease and Alzheimer's disease (AD), it is difficult to speculate about the causal link between these two affections, but as pointed out by Polidori et al. [31] and in accordance with the present findings, we think that a systematic neuropsychologic testing of older patients with heart failure should be processed in order to identify those with early cognitive impairment and promptly establish traditional therapies such as angiotensin converting enzyme inhibitors, digoxin, or beta-blockers. In addition, the neuropsychologic assessment in cardiovascular patients is also probably fundamental to disclose conditions potentially favoring the onset of cognitive impairment such as depression. From a clinical point of view, multidisciplinary approach is necessary to deal with the complexity of the cognitive consequences of cardiovascular disease and we think that in these patients, management schemes should also include exercise training programs (see, e.g., Boucard et al. [32]) as well as patient and caregiver education. From a more experimental point of view, despite the small number of participants, the present results demonstrate an effect of both normal aging and cardiovascular disease on selective attention processes and on functional organization for this kind of task. These preliminary findings underline the need to study in larger groups how aging and cardiovascular disease may interact to asymmetrically modify the cerebral functioning and in this way the underlying cognitive processes. This could include the use of brain perfusion SPECT imaging in order to search for anatomical correlates of vascular cognitive impairments [33]. Our present preliminary findings also indicate that research in the field of cognitive function in the elderly should take into account the (medical and more specifically) cardiac history of the experimental population to be studied.

## Figures and Tables

**Table 1:** Reaction times in ms, (standard deviations) for big targets presented alone or small targets surrounded by flankers, in each hemifield.

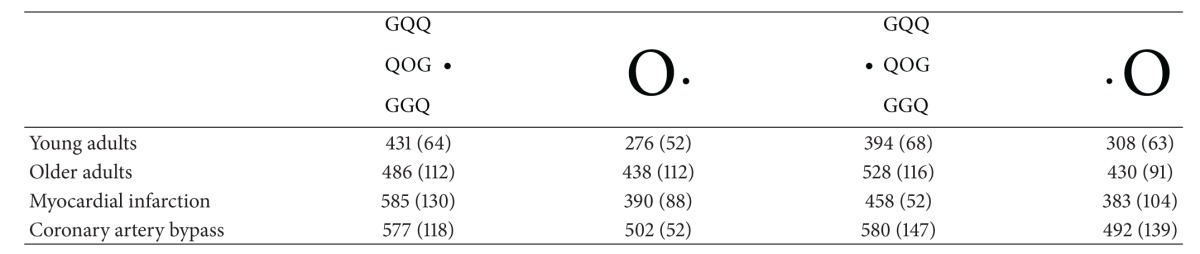

**Table 2:** Accuracy (average number of correct responses and standard deviations) for big targets presented alone or small targets surrounded by flankers in each hemifield.